# A p38 Substrate-Specific MK2-EGFP Translocation Assay for Identification and Validation of New p38 Inhibitors in Living Cells: A Comprising Alternative for Acquisition of Cellular p38 Inhibition Data

**DOI:** 10.1371/journal.pone.0095641

**Published:** 2014-04-17

**Authors:** Roman Anton, Silke M. Bauer, Peter R. W. E. F. Keck, Stefan Laufer, Ulrich Rothbauer

**Affiliations:** 1 NMI Natural and Medical Sciences Institute at the University of Tuebingen, Reutlingen, Germany; 2 Pharmaceutical Biotechnology, University of Tuebingen, Tuebingen, Germany; 3 Institute of Pharmacy, University of Tuebingen, Tuebingen, Germany; Temple University, United States of America

## Abstract

The fundamental role of p38 mitogen-activated protein kinases (MAPKs) in inflammation underlines their importance as therapeutic targets for various inflammatory medical conditions, including infectious, vascular, neurobiological and autoimmune disease. Although decades of research have yielded several p38 inhibitors, most clinical trials have failed, due to lack of selectivity and efficacy *in vivo.* This underlines the continuous need to screen for novel structures and chemotypes of p38 inhibitors. Here we report an optimized MK2-EGFP translocation assay in a semi-automated image based High Content Analysis (HCA) system to screen a combinatorial library of 3362 proprietary compounds with extensive variations of chemotypes. By determining the levels of redistribution of MK2-EGFP upon activation of the Rac/p38 pathway in combination with compound treatment, new candidates were identified, which modulate p38 activity in living cells. Based on integrated analysis of TNFα release from human whole blood, biochemical kinase activity assays and JNK3 selectivity testing, we show that this cell based assay reveals a high overlap and predictability for cellular efficacy, selectivity and potency of tested compounds. As a result we disclose a new comprehensive short-list of subtype inhibitors which are functional in the low nanomolar range and might provide the basis for further lead-optimization. In accordance to previous reports, we demonstrate that the MK2-EGFP translocation assay is a suitable primary screening approach for p38-MAPK drug development and provide an attractive labor- and cost saving alternative to other cell based methods including determination of cytokine release from hPBMCs or whole blood.

## Introduction

The mammalian p38 mitogen-activated protein (MAP) kinases are belong to an evolutionary highly conserved family of serine/threonine kinases which transduce extracellular signals in response to inflammation and external stress to the nucleus and thereby enabling cells to respond to environmental stimuli. Their central role in inflammatory signal transduction has been closely related to inflammation-caused diseases, including autoimmune diseases (e.g. rheumatoid arthritis), neurobiological disorders (e.g. epilepsy, Alzheimer’s disease), and other types of diseases like atherosclerotic disease progression [1]–[4].

**Figure 1:**
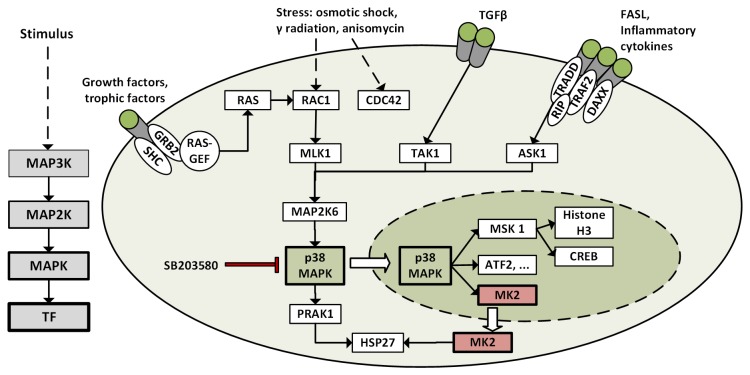
Activation of p38 MAPK in response to certain stimuli. Dashed lines refer to one or more stimuli. Bold arrows refer to translocation of the kinase in response to activation by upstream kinases.

p38 kinases are activated by abiotic stressors, e.g. DNA damage (UV light, anisomycin), heat, hyperosmotic shock, wear stress, oxidative stress or by chemical induction including pro-inflammatory stimuli (cytokines, LPS), interleukin 1 or tumor necrosis factor (TNF) α. Activation occurs through a dual phosphorylation of Thr180 and Tyr182 mediated by MAP2K3/MKK3 or MAP2K6/MKK6. Upon activation p38 kinases phosphorylate and activate transcriptions factors or other downstream kinases including MapKap2/MK2, MapKap3/MK3 or MSK1 which subsequently activate components involved in mRNA stabilization or gene transcription. This results in the induction of immediate early genes in response to stress or mitogenic stimuli such as interleukin-1 and TNFα [2], [5], [6] (**Figure 1**).

The MAP kinase family consists of three subfamilies that include the extracellular signal-regulated kinases (ERKs), the c-Jun N-terminal kinases (JNKs), and p38 kinases. There exist four p38 isoforms p38α, p38β, p38γ and p38δ which show differences in the activation modes, tissue expression and substrate preferences [7], [8]. The ATP-binding site is highly conserved across related members of specific kinase subfamilies. While p38α and p38β show 83% sequence identity other members of nearby kinase families like JNK3 still share 51% identity in their primary sequence. Therefore gaining selectivity between p38α/β and JNK3 is very challenging. A promising approach for gaining p38 inhibitor selectivity over JNK3 is taking advantage of the so-called “gate keeper” residues, which are located inside the ATP pocket at the entrance of the “hydrophobic region I”.

The historical development of anti-inflammatory drugs and the resultant p38α inhibitors started in the late 1970s and early 1980s with SKF86002, an imidazothiazole scaffold which was suggested to act as a substrate competitive inhibitor [9]. Originally described as a cytokine suppressive anti-inflammatory drug (CSAID) with potent anti-inflammatory profile due to a dual mode of action through the dual inhibition of cyclooxygenase and lipoxygenase, SKF86002’s action as p38 MAPK inhibition could be shown by [10]. Further research revealed more pyridinylimidazoles analogues including the most famous representative SB203580 of this series [11]. All these “first generation” p38 MAPK inhibitors suffer from structure based toxicity mainly of the imidazole via cytochrome-P450 enzymes and poor selectivity. Until today many more chemotypes and isoforms have been discovered targeting the ATP binding pocket or an allosteric site in a more selective fashion [12]–[16]. Despite promising results *in vitro* and *in vivo*, many clinical trials showed poor outcome, primarily due to adverse effects, lack of efficacy and only modest clinical activity on the target indication [3], [17]–[19]. Hence, there is a continuous search for new subtypes of p38 inhibitors providing improved *in vivo* efficacies, reduced side effects and better therapeutic potentials [20].

In order to identify new compounds which modulate the secretion of pro-inflammatory cytokines activated human peripheral blood monocytes (hPBMCs) or assay systems using human whole blood in general are part of every standard screening procedure. However, this strategy is more indirect in terms of molecular mechanism as transcription and release of cytokines are the last steps of a signaling cascade that involves p38 upstream-activation. Moreover, the use of costly, pre-tested blood from human donors is required. Although these *ex vivo* assays are still very important to predict *in vivo* efficacy and therapeutic potential of isolated compounds, they entail severe disadvantages for primary high throughput screening campaigns including high variability, costs, laboriousness, incapability of automation with regard to HTS and indirectness.

**Figure 2:**
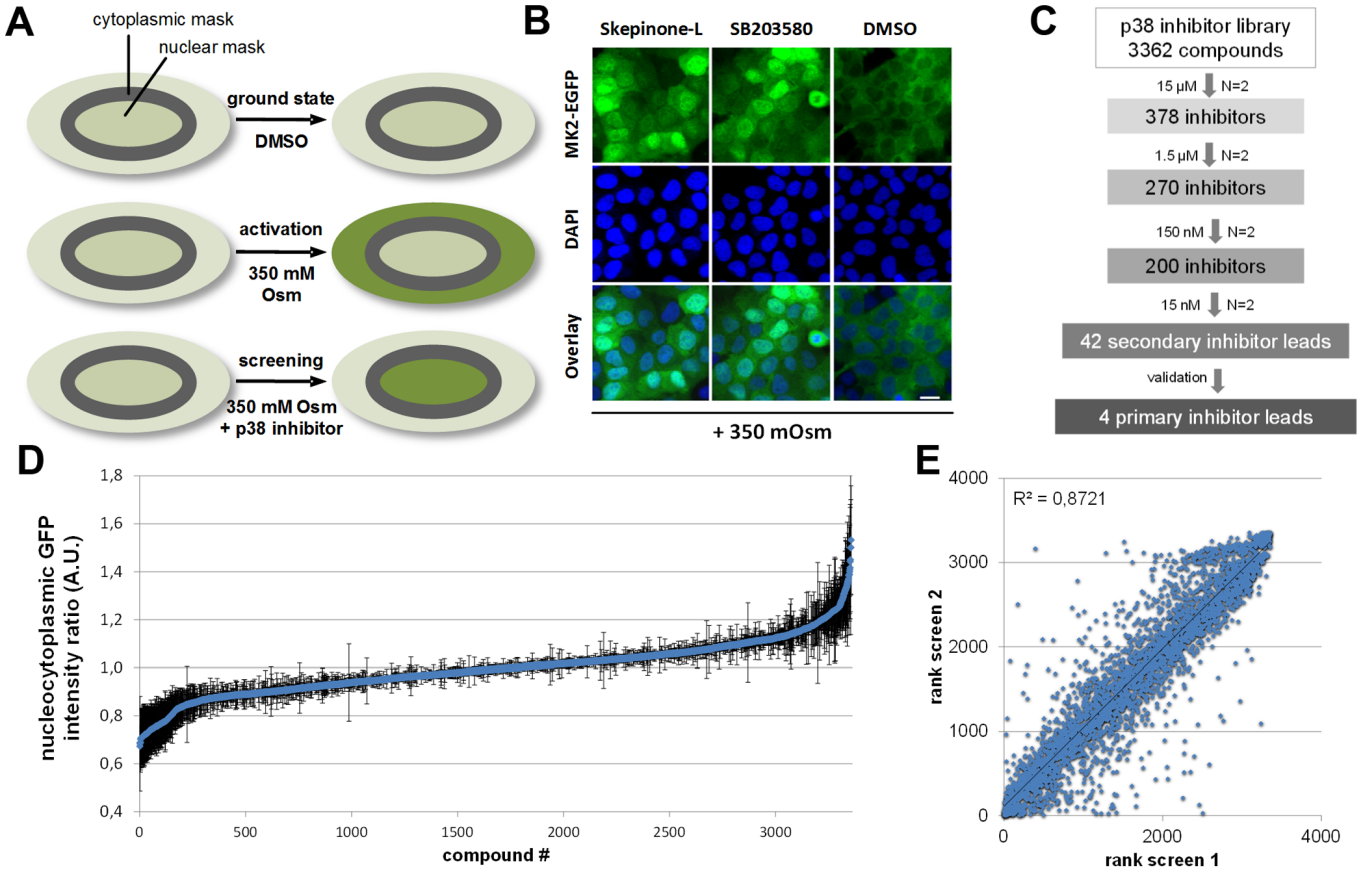
Cellular MK2-EGFP translocation assay to screen for p38 inhibitors. (A) Schematical outline of MK2-EGFP translocation assay. Nuclear DAPI segmentation facilitates determination of the nucleocytoplasmic MK-2-EGFP fluorescence intensity ratio (grey ring). In the ground state U2OS cells stably expressing MK2-EGFP shows a slightly higher fluorescent intensity in the nucleus compared to the cytoplasm. For activation of the Rac/p38 pathway cells were subjected to hyperosmotic stress (175 mM NaCl/350 mOsm) leading to a rapid relocation of MK2-EGFP from the nucleus to the cytoplasm. Co-treatment with p38 inhibitors abolishes this redistribution, which is visualized in high content analysis. Determination of fluorescent intensities provides a cellular read-out with appropriate z’ values above 0.5. (B) Representative pictures of activated U2OS MK2-EGFP cells either non-treated (DMSO) or treated with p38 inhibitors Skepinone-L or SB203580, scale bar 20 µM. (C) Workflow of the p38 inhibitor screen, validation procedures and number of identified compounds (D) Sorted results of the entire primary p38 inhibitor screen represented as average cellular nucleocytoplasmic fluorescence intensity ratios reflecting the distribution of MK2-EGFP (E) Rank comparison of the first and second biological replica of the primary screen at 15 µM showing the reproducibility of the results. R^2^ of >0.85 indicate reliability for further cherry-pick listing.

To avoid disadvantages of the labor-intensive and non-automatable whole blood assay for acquisition of cellular data there is a need for alternative cell based techniques. For detection of p38 activity the here reported MapKap2 (MK2) - translocation assay is a suitable primary screening approach. This cell based assay (Figure 2 A) involves activation of Rac/p38 pathway followed by the phosphorylation of p38 MAPK and subsequent binding and phosphorylation of its nuclear substrate MapKap2 (MK2). Binding of p38 MAPK to MK2 masks the nuclear localization site (NLS) and induces a structural change exposing the nuclear export signal (NES) of MK2 [21], [22]. As a consequence the translocation of MK2 from the nucleus to cytoplasm can be visualized using a fluorescently labeled MK2-EGFP construct. Recently it has been shown that this translocation is invertible by several p38 inhibitors [23]–[25]. We generated a new sensitive U2OS cell line stably expressing MK2-EGFP and we induced p38 activity simply via hypertonicity. Redistribution of MK2-EGFP upon activation of the Rac/p38 pathway yielded different nucleocytoplasmic ratios of the fluorescently labeled MK2-EGFP construct. This information allows us to draw conclusions about the potency of p38 activity modulating compounds.

Applying this p38 substrate-specific MK2-EGFP translocation assay in a semi-automated image based High Content Analysis (HCA) system, we identified new lead sub-chemotypes down to nanomolar potency. For this purpose, we took advantage of a valuable compound library containing various chemotypes and modifications, which was screened for potent p38 inhibitors using a living cell system. Selected compound specificities were compared to data from a biochemical p38α kinase assay and a whole blood assay for TNFα-release. Results were merged in a new short-list of leads with high potency, comprising two families: dibenzosuberones and imidazoles. The integrated data analysis provides comprehensive -functional information of the ATP pocket binder library. Validation of identified top leads in a c-jun/JNK3 assay revealed their specificity for p38, while cellular viability assays gave no indication for cytotoxic effects. In our study we demonstrate that the combination of high throughput biochemical testing, semi-automated live cell screening followed by a sensitive whole blood assay offers a versatile and effective approach to generate and validate a new short-list of intracellular functional p38-inhibitors which could be now subjected to lead optimization.

## Materials and Methods

### Compound Preparation and Handling

All library compounds were originally dissolved in DMSO yielding concentrations of 10 mM and aliquoted into 96-well plates. These master plates were used to generate working stock plates of compounds at 150 µM concentration in Hank’s Buffered Saline Solution and 10% DMSO. The compounds were added to screening plates as a 10x solution yielding a 15 µM final concentration. The final concentration of DMSO was 1%. For further analysis of lower concentration (1.5 µM, 150 nM, 15 nM and 1.5 nM) selected compounds were diluted from the working plates accordingly.

### Whole Blood Assay

For determination of inhibitor potency of the compounds by detection of TNFα release in human whole blood an *in vitro* assay using human whole blood stimulated with LPS for TNFα synthesis is a reliable test system for inhibitors of cytokine synthesis, especially for p38 MAP kinase inhibitors as TNFα synthesis is mainly mediated by p38 MAP kinase. Therefore, the synthesis and release of proinflammatory cytokines in human whole blood from a healthy donor is stimulated using a LPS solution (1 µg/mL, Sigma-Aldrich, Germany) after preincubation of inhibitor dilution (as described in [26]). After 2.5 hours of incubation in a 5% CO_2_ incubator, the stimulation is stopped and plasma is obtained by centrifugation of the whole blood, followed by TNFα readout from the plasma using a commercially available TNFα ELISA kit (R&D Systems, Germany). The blood sample was obtained with the written informed consent of the donor to participate in this study. The ethics committee (Ethik-Kommission der Medizinischen Fakultät und am Universitätsklinikum Tuebingen) state that an ethical statement is not mandatory when the researchers use their own blood/plasma for experiments. The german statutes and the Declaration of Helsinki are not aimed at experiments using samples provided by the principal investigator him or herself. In the opinion of the committee, the Declaration of Helsinki is designed to protect the patient’s interests and to avoid abuse of the patient’s material.

### Biochemical p38α and JNK3 Activity Assays

Kinase activity assays were used for identification of p38α MAPK and JNK3 inhibitors. The experimental settings and screening procedures are previously described by Goettert et al. [27], [28].

Briefly, the ELISA assay was performed using 96-well plates (MaxiSorp, NUNC, Germany) by measuring the activity of p38α MAPK after incubation with a candidate inhibitor. Therefore, the phosphorylation degree of activation transcription factor 2 (ATF-2) is detected by a monoclonal peroxidase conjugated antibody in a dilution of 1∶5000 (Sigma-Aldrich, Germany). This monoclonal anti-phospho-ATF-2 (Thr69/71) peroxidase-conjugated antibody reacts specifically with dually phosphorylated ATF-2, detecting neither the mono- nor the unphosphorylated kinase substrate. ATF- 2 is a natural substrate of the p38 MAPKs and JNK3, and its phosphorylation is inversely correlated with the inhibitor potency.

### Cell Culture

U2OS cells (# HTB-96, ATCC, USA) were cultivated according to standard protocols. Briefly, growth media contained DMEM (high glucose, pyruvate, 10% fetal calf serum; PAA, Germany), supplemented with L-Glutamine and antibiotics. Cells were trypsinized for passaging and cultivated at 37°C in a humidified chamber with a 5% CO_2_ atmosphere. Stable cell lines were maintained in DMEM supplemented with 10% fetal calf serum, gentamycine at 50 µg/mL (PAA, Germany) and G418 at 1 mg/mL (PAA, Germany).

### Generation of a Stable U2OS MK2-EGFP Cell Line

A mammalian expression vector comprising an N-terminal GFP-tagged version of the full length human MK2 open reading frame under the control of the CMV promotor (kindly provided by J. Gregor, LMU-Biocenter) was transfected using Lipofectamin 2000 (Life Technologies, Germany) into U2OS cells followed by 4 week clonal propagation under 1 mg/mL G418 (PAA, Germany) selection. Cells were serial diluted in 96-well plates and single clones were obtained. Clones with appropriate transgene expression levels were selected and MK2-EGFP expression in transgenic clones was confirmed by SDS-PAGE and immunoblotting using an antibody against GFP (3H9, ChromoTek, Germany). Several clones were tested for the MK2-EGFP redistribution upon p38 activation in hyperosmotic media (10% FCS, DMEM, P/S, L-Glut, 175 mM NaCl; PAA, Germany) and its blockage by the p38 inhibitor SB203580 (Life Technologies, Germany) (Figure S1). Finally one clone (U2OS-MK2-EGFP-clone-RA#2) was chosen for further experiments.

### Determination of Z′factors

Z’ factors were calculated according to the formula: 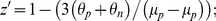


p: positive control and n: negative control, θ, standard deviation, µ, mean [29].

### High Content Screening

Screening was performed in biological duplicates on different days and different cell line passages. For cellular screening ∼ 12.500 cells were seeded into 96-well clear tissue-culture micro-plates (Bio-One µClear, Greiner, Germany) and grown until 90% confluence. The Rac/p38 MAPK pathway was activated by exchanging normal growth media with hyperosmotic media. Immediately, compounds were added yielding a final concentration of 15 µM. Plates were mixed thoroughly and incubated at 37°C and 5% CO_2_ in a humidified chamber atmosphere. After 80–90 minutes the treatment was stopped by disposing the media. Subsequently cell were fixed using 3% PFA/PBS in 1x PBS at pH 7.4 for 15 minutes and washed with PBS and PBST (0.05% Tween-20) following nuclear DNA staining using 150 ng/mL DAPI in 1xPBS/35 µg/mL RNAseA. Finally, plates were transferred in an automated cytomat incubator (Thermo Scientific, Germany) combined with a robotic plate handling device (PAA, UK) and subjected to automated image acquisition.

### HCA Image Acquisition and Analysis

Images of EGFP and DAPI fluorescent cells were acquired using an Image Xpress micro XL microscope (Molecular Devices, Germany). At least 4 sites were acquired per well using a 20x Super Pan fluor ELWD cc 0–2 mm objective and FITC (482/35) and DAPI (377/50 nm) filter sets with exposure times of 4 ms for DAPI and 110 ms for FITC channel. On average 400 cells per well were imaged, allowing reliable statistical measures and quantification. To measure the ratio of nucleocytoplasmic fluorescent intensities a nuclear compartment surrounded by a peripheral DAPI-negative cytoplasmic ring was defined using the translocation-enhanced application module based on a DAPI nuclear mask with following Vernier-adjustments: compartment image: DAPI; translocation probe image: FITC; compartment algorithm: standard; approximate width: 13 µm; intensity above local background: 656 to 65535 gray-levels; minimum area: 60; maximum area: 355; auto-separate touching objects; inner region distance: 0.5 µm; outer region distance: 0.5 µm; outer region width: 2 µm; background estimation method: auto-constant; classify positive> = 1.

Automated image analysis was carried out using the MetaXpress software (Molecular Devices, Germany). Fluorescence intensity ratios were calculated using the average of the cellular MK2-EGFP fluorescence intensity (Cell: nuclear inner/outer mean intensity) of all images/sites per well. p38 activation was defined as positive when the nucleocytoplasmic MK2-fluorescence intensity ratio was below 0.7. For further analysis, the average of the mean of the entire plate was calculated and used for internal plate normalization. For quality assessment of the screening the Log-Z-score was determined [30]. Obtained values were calculated on the normalized nucleocytoplasmic MK2-fluorescence intensity ratio (average of the mean) of respective data-points. Treatment with Skepinone-L and SB203580 were defined as positive controls, while treatment with DMSO was defined as negative control.

### Hit Validation

Validation of 368 cherry-picked leads was performed in biological duplicates in four 96 well plates in low FCS-level hyperosmolar growth media (3% FCS, DMEM, P/S, L-Glut, 175 mM NaCl). Selected compounds were tested in three concentrations: 1.5 µM, 150 nM, and 15 nM. Image analysis and result calculations were performed as described.

### p38 Specificity Testing in Phospho-c-jun/JNK Assay

Compound specificity towards p38 and JNK were analyzed in a secondary JNK activity assay detecting phosphorylated c-Jun (Ser63) as a substrate of JNK. This assay could be easily multiplexed with the MK2-EGFP assay as described by Henriksen et al. [31]. Briefly, stimulated U2OS-MK2-EGFP-clone-RA#2 cells were fixed in 96-well plates with 4% PFA/PBS, blocked and permeabilized with protein-free blocking media (Pierce, USA) in TBS with 0.1% Triton X-100. Samples were incubated overnight at 4°C with an antibody directed against phospho-c-Jun Ser63 (#9261, Cell Signaling Technology, USA) diluted 1∶150 in TBST followed by detection with an Alexa647-goat-α-rabbit (Life Technologies, Germany). After washing procedures cells were subjected to image acquisition and automated image-analysis as described. DMSO served as a negative control, as positive control SP600125 - an anthrapyrazolone type JNK inhibitor with a 300-fold greater selectivity over p38 - was used [32].

### Cellular Viability Assay

The cellular viability assay was performed according to standard procedures (for details see AlamarBlue manual #DAL1100, Life Technologies, Germany). Briefly, ∼ 15.000 cells of the U2OS MK2-EGFP clone-RA#2 cell line were plated in 96 well µclear plate and grown in growth media until sub-confluency. Subsequently, cells were treated with compounds in indicated concentrations for 24 h at 37°C in a humidified chamber containing 5% CO_2_. After compound treatment the amount of living cells was monitored microscopically and cell-viability was determined using the AlamarBlue assay. For this purpose, resazurin was added freshly to the growth media devoid of phenol red as described. The media premixed with the resazurin substrate was pre-warmed to 37°C and 200 µL were added to the pre-treated cells in the 96-well plate. Following incubation at 37°C, 5% CO_2_ for 4 h the fluorescence of the media was measured in a PHERAstar photometer (BMG Labtech, USA) with the filter sets: Ex: 540nm/Em: 580nm.

## Results and Discussion

### Description of the p38 Inhibitory Compound Library

A small-molecule library comprising 3362 compounds enriched for p38 MAPK and JNK3 inhibitors based on heterocyclic five and six membered rings and fused heterocyclic ring systems was developed in the laboratory of Prof. Laufer. Approximately 2500 compounds are mainly based on substituted imidazole scaffolds, but also on substituted isoxazoles, pyrazoles, pyrroles, pyrimidines, quinolones, azaquinolones, thiazolotriazoles and dihydropyranopyrazoles. To exploit characteristics that are unique to p38α MAP kinase such as the hydrophobic region I, the library was supplemented with substituted dibenzosuberones structures to generate more selective inhibitors. Those compounds possess the required rigidity to address the flexible structures of the kinases, which might induce conformational changes during activation. As a result, the other main part of the library consists of approximately 500 oxepinones and dibenzosuberones such as Skepinone-L and its analogues. In summary, the library tested here comprises various combinations of functional groups and chemo-types yielding sub-isoform arrays of diverse pharmacological properties (S. Laufer, pers. communication).

### Cell Based Screening System of p38 Inhibitors

To screen for intracellular functional p38 kinase inhibitors we chose a recently described cellular MK2-EGFP translocation assay [33]. In a first step we generated a new U2OS MK2-EGFP transgenic cell line. We selected one cellular clone with appropriate MK2-EGFP expression to visualize the MK2-EGFP redistribution from the nucleus to the cytoplasm. For activation of the Rac/p38 pathway cells were treated either with anisomycin or exposed to hyperosmotic stress by adding different concentrations of NaCl to the growth media (Figure S1). Activation with 175 mM NaCl (350 mM Osm) induces no observable side effects and has no impact on the cellular viability compared to treatment with anisomycin (data not shown). Time lapse analysis indicated a complete translocation of MK2-EGFP from the nucleus within 30 min after activation of the Rac/p38 pathway upon hyperosmotic treatment. p38 activation was defined as positive when the nucleocytoplasmic ratio was below 0.7.

For initial testing we use SB203580 and the recently identified highly selective p38 inhibitor Skepinone-L [34]. Our results show that treatment of the cells with 10 µM of each compound abolishes nuclear export of MK2-EGFP in our cellular model significantly (Figure S2, Movie S1). Next we tested appropriate screening conditions and selected following ones: usage of a stable clone of a medium-level expressing U2OS MK2-EGFP cell line, cellular treatment at high confluence (>90%), activation of Rac/p38 signaling with median hyperosmotic stress (350 mM Osm), addition of inhibitors during activation for 75–90 min. By measuring the nuclear/cytoplasmic fluorescent intensity ratio of pre-activated cells treated either with DMSO (negative control) or the p38 inhibitor SB203580 (positive control) we were able to determine appropriate z’ factors above 0.5 [29]. To exclude short-time effective inhibitors, incubation times and screening endpoints were set up to 75–85 minutes.

We validated the assay by testing the inhibitory effect of SB203580 and Skepinone-L on MK2-EGFP translocation (Figure 2 B). For nuclear segmentation we combined the dynamic live cell assay with a DAPI/DNA staining protocol to determine the nuclear/cytoplasmic distribution of MK2-EGFP. Automated image acquisition and image based data analysis show an accurate quantification of the nucleocytoplasmic ratio of MK2-EGFP fluorescence after compound treatment (Figure S2).

### Screening and Validation of Compound Library

The initial screening was performed in biological duplicates using compound concentrations of 15 µM. At least 500 cells per treatment condition were monitored to achieve sufficient statistical robustness. A flow-chart of the screening strategy summarizes the results of the primary screen and subsequent follow-up validation rounds and cherry-pick selection (Figure 2 C).

After the first round of screening we identified approximately 300–400 highly effective p38 inhibitors. Figure 2 D depicts the results of the primary screen (N = 2) represented as mean nucleocytoplasmic intensity ratios (for screening data see Table S1). Reproducibility showed good confidence between biological replica 1 and 2, as indicated by R^2^>0.85 in the rank-rank comparison (Figure 2 E). For detailed results of the initial MK2-EGFP translocation screen see Table S1.

**Figure 3:**
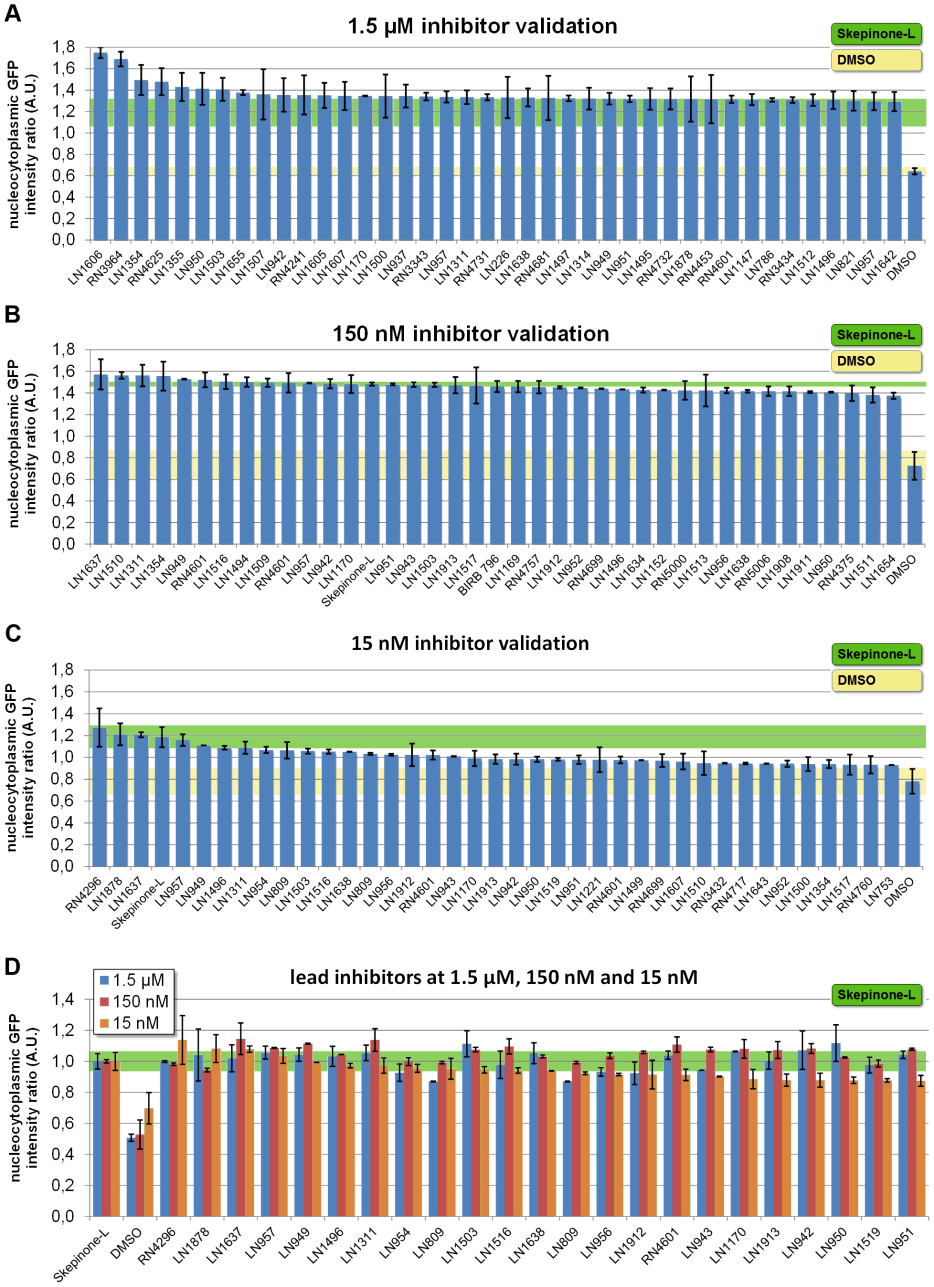
Validation of the screening results using the MK2-EGFP translocation assay at decreasing compound concentrations. Confirmation of p38 inhibition of selected 378 cherry-picks at (A) 1.5 µM, (B) 150 nM, and (C) 15 nM. Green stripe: Skepinone-L top level bench mark of inhibition of nuclear export of MK2-EGFP. Yellow strip: nuclear intensity in activated cells treated with DMSO. Top 40 inhibitors are shown, including Skepinone-L and negative control (DMSO). (D) Comparison of lead inhibitor activity at 1.5 µM, 150 nM and 15 nM. Nucleocytoplasmic fluorescent ratio after treatment with Skepinone-L is set to 1. For direct comparison nucleocytoplasmic fluorescent ratios induced by all validated compounds are normalized to Skepinone-L.

Subsequently we selected compounds inducing nucleocytoplasmic fluorescent ratios higher than 1 (>1) for further analysis at different concentrations. For validation of the selected inhibitors we performed three screening rounds using following concentrations: 1.5 µM, 150 nM, and 15 nM (each in biological duplicates, N = 2). To include compounds which exhibit serum albumin binding we reduce the amount of fetal calf serum (FCS) from 10% to 3% in the growth media conditions. Validation rounds of 378 selected compounds consistently extracted the most potent inhibitors with increasing stringency from medium to very low concentrations. The top 41 validated inhibitors at 1.5 µM - including Skepinone-L – are shown in Figure 3 A. Notably, the first validation round of 378 selected compounds at 1.5 µM revealed high validation rates of above 70% of the hit list obtained in the primary screen. The second validation round of the 378 hits at 150 nM still yielded approximately 200 compounds with strong p38 inhibition indicated by nucleocytoplasmic ratios of above 0.95 compared to DMSO (0.65). Figure 3 B highlights the top 41 inhibitors revealing 23 substances with comparable activity to Skepinone-L (nuc/cyt ratios: 1.37–1.48). After lowering the compound concentration to 15 nM we still re-identified 27 compounds which significantly block translocation of MK2-EGFP indicating a high potency to inhibit p38 MAPK (Figure 3 C).

Finally we tested the 23 most potent compounds in three concentrations (1.5 µM, 150 nM, 15 nM) and two biological replicates. To directly compare the inhibitory effect of the individual compounds with the benchmark levels of Skepinone-L the nucleocytoplasmic ratios obtained for Skepinone-L was set to 1 for each tested concentration and MK2-EGFP distribution ratios detected for any of the tested compounds were normalized accordingly (Figure 3 D). The results confirmed an outstanding potency of Skepinone-L to inhibit p38 at very low concentrations (15 nM). However, some compounds could slightly exceed the efficacy of Skepinone-L in the MK2-EGFP translocation assay at higher concentrations (15 µM–150 µM). Here we identified RN4296 as the most potent compound towards p38 inhibition (Figure 3 A–D) in the translocation screen.

### Hit Validation in Whole Blood Assays and Biochemical Activity Assays

In a next step we performed whole blood assays and biochemical p38 activity assays to integrate our results of the cell based screen with data from conventional p38 inhibitor screenings. Therefore we selected 35 of the most potent inhibitors from the MK2-EGFP translocation assay (TA) and tested them in human whole blood assays (WBA) on the release of TNFα after stimulation with lipopolysaccharide (LPS) (Table 1). Additionally we performed biochemical activity assays (BAA) determining the IC50s of the selected compounds according to their potencies to inhibit purified p38 MAPK. As a functional read out we determined the inhibition of phosphorylation of ATF-2 (Thr69/71) as described previously (Table 1) [27]. All experiments were performed in three biological replicates and the results of both assays are summarized in Table 1.

**Table 1:** Comparative data table of top 35 inhibitors selected from MK2-EGFP translocation assay (TA) determining the nuclearcytoplasmic ratio at 15 nM compound concentration (N = 3), whole blood assay (WBA) determining TNFα release after LPS stimulation (N = 3) and the p38 MAPK activity assay (BAA) determining the IC50 on p38 MAPK activity detected by inhibition of phosphorylation of ATF2 (Thr69/17) (N = 3).

*Lab #*	nuc/cyt ratio at 15 nM ± SD	WBA TNFα IC50 [nM] ± SD	BAA p38 IC50 [µM] ± SD	Citation
***Skepinone-L***	1,1851±0,0926	0,059±0,038	0,0067±0,0022	[14]
***RN4296***	1,288±0,2483	n.d.	0,0037±0,0006	
***LN1878***	1,211±0,1416	n.d.	0,0019±0,0001	
***LN1637***	1,206±0,0326	0,006±0,001	0,0005±0,0001	[26]
***LN957***	1,158±0,0756	0,129±0,018	0,0030±0,0005	[36]
***LN949***	1,111±0,0012	0,173±0,037	0,0117±0,0018	[36]
***LN1496***	1,088±0,0231	0,037±0,009	0,0032±0,0006	[26]
***LN1311***	1,088±0,0781	0,106±0,033	0,0011±0,0000	[26]
***LN954***	1,068±0,0406	0,182±0,150	0,0057±0,0012	[36]
***LN809***	1,064±0,1062	0,047±0,010	0,0101±0,0022	[14]
***LN1503***	1,057±0,0332	0,221±0,163	0,0013±0,0002	[26]
***LN1516***	1,052±0,0282	0,037±0,013	0,0015±0,0001	[26]
***LN1638***	1,051±0,0022	n.d.	0,0054±0,0008	
***LN956***	1,023±0,0125	0,035±0,004	0,0023±0,0007	[36]
***LN1912***	1,023±0,1459	0,156±0,108	0,0290±0,0026	
***RN4601***	1,021±0,0586	0,123 (n = 1)	0,0041±0,0002	[37]
***LN943***	1,009±0,0036	0,183±0,016	0,0050±0,0013	[36]
***LN1170***	0,990±0,0992	0,084±0,033	0,0118±0,0016	[36]
***LN1913***	0,983±0,0612	n.d.	0,0029±0,0002	
***LN942***	0,983±0,0715	0,115±0,013	0,0029±0,0003	[36]
***LN950***	0,982±0,0335	0,021±0,004	0,0118±0,0017	[36]
***LN1519***	0,981±0,0179	0,083±0,040	0,0022±0,0003	[26]
***LN951***	0,978±0,0556	0,168±0,032	0,0173±0,0034	[36]
***LN1221***	0,977±0,1603	1,722±0,646	0,0159±0,0023	[38]
***LN1499***	0,973±0,0007	0,573±0,198	0,0978±0,0086	[26]
***RN4699***	0,971±0,0821	0,130±0,006	0,0123±0,0008	
***LN1607***	0,961±0,1018	0,050±0,024	0,0035±0,0001	[26]
***LN1510***	0,947±0,1522	0,019±0,016	0,0098±0,0037	[26]
***RN4717***	0,943±0,0121	0,614±0,250	0,0057±0,0004	[37]
***LN1643***	0,942±0,0027	n.d.	0,2266±0,0354	
***LN952***	0,941±0,0411	0,153±0,032	0,0099±0,0046	[36]
***LN1500***	0,938±0,0898	0,026±0,004	0,0002±0,0000	[26]
***LN1354***	0,938±0,0525	0,029±0,006	0,0025±0,0002	[26]
***LN1517***	0,933±0,1306	0,047±0,023	0,0045±0,0008	
***RN4760***	0,932±0,1131	n.d.	0,0383±0,0102	[39]

The results showed that with the MK2-EGFP translocation assay (TA) we have identified those compounds, which are also selected as potent inhibitors of p38 MAPK in established screening assays (Table 1). Detailed analysis reveal that some of the identified compounds like LN1637 exhibit a high inhibitory potency in all tested screening approaches (TA: 15 nM; WBA, IC50∶6 nM; BAA, IC50∶5 nM). In contrast, other substances like LN1499 (TA: 15 nM; WBA, IC50∶0.5 µM; BAA, IC50∶98 nM) have a strong impact on MK2-EGFP translocation in living cells but show less activity in the other test systems. However, these variations might be due to the different availability or stability of the compounds under different screening conditions. Although the potency of identified inhibitors varies in the different screening formats, the results show an overall comparable identification of a new short list of high potent inhibitors.

### Structural Analysis of Identified Compounds

**Figure 4:**
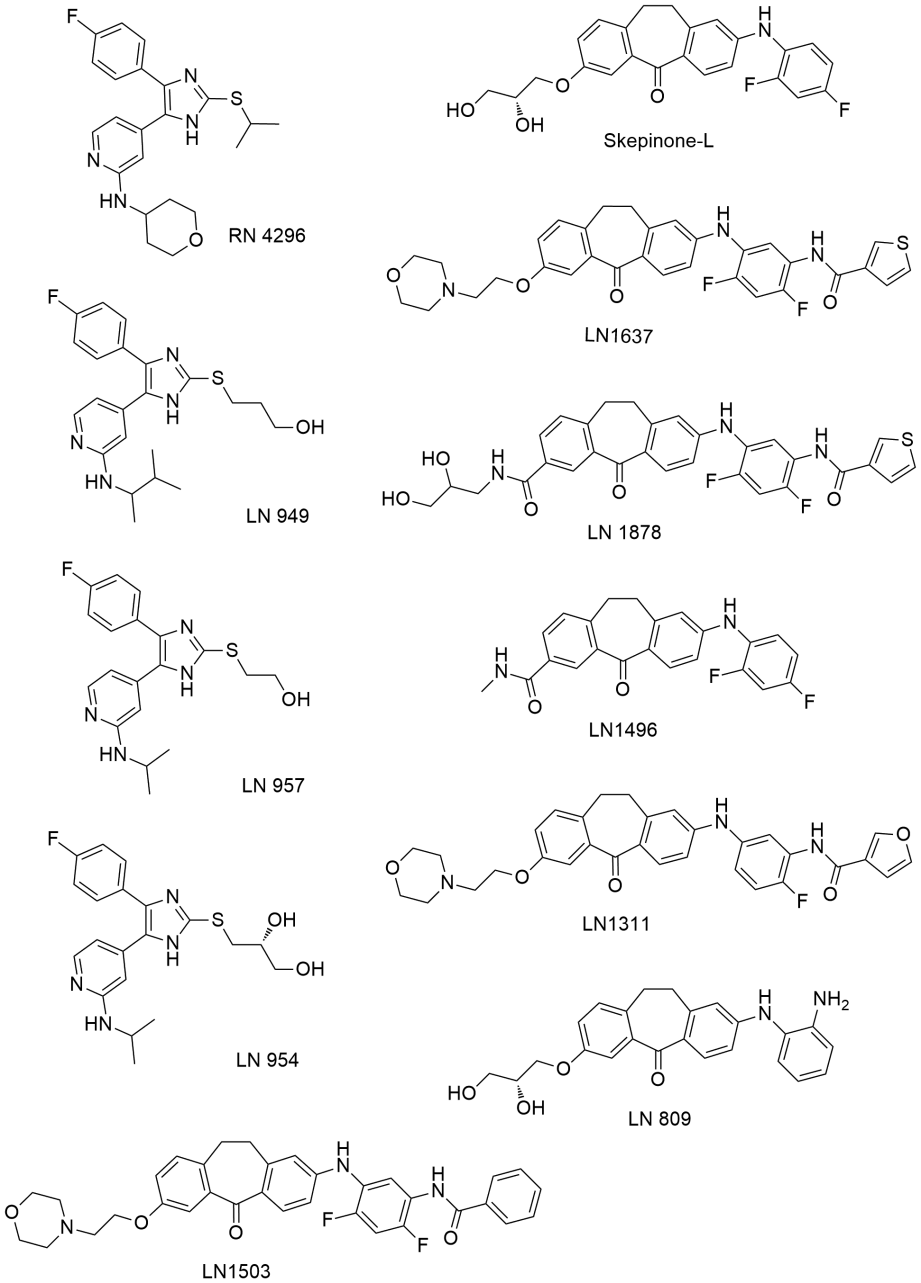
Structures of identified top 10 inhibitors.

An analysis of the chemical structures of the 10 most potent inhibitors identified from the MK2-EGFP translocation screen revealed that six compounds belong to the class of dibenzosuberones. From the structural analysis we cannot conclude any structural features or chemical substitutions which might influence the intracellular availability or functionality. Interestingly we identified four compounds - including the most potent compound RN4296 - as members of the first generation imidazole-based structural class (Figure 4). This demonstrates that the cell based translocation assay allows an unbiased selection of highly active inhibitors from different chemical scaffolds. However none of the substituted isoxazoles, pyrazoles, pyrroles, quinolones, azaquinolones, thiazolotriazoles and dihydropyranopyrazoles were identified, fitting nicely to the absence of these compound classes in clinical trials.

**Figure 5:**
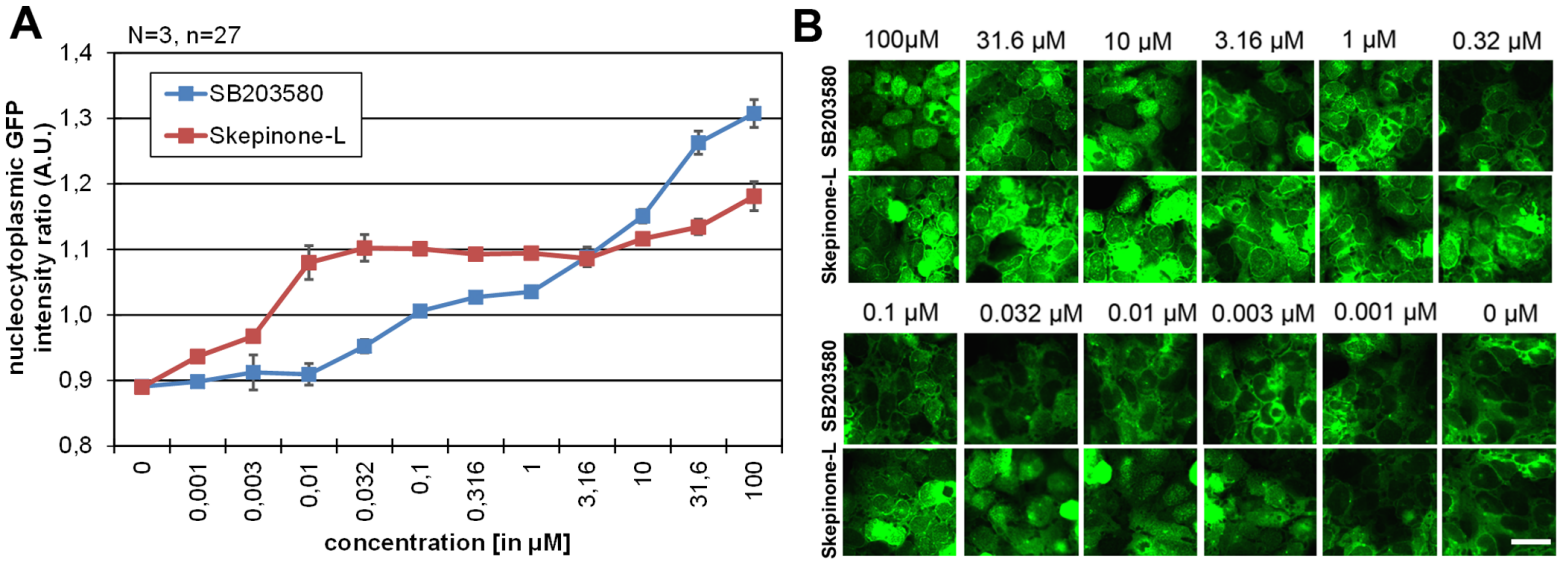
Comparison of imidazole derived inhibitor SB203580 with the dibenzosuberone derived inhibitor Skepinone-L in the MK2-EGFP translocation assay. (A) Nucleocytoplasmic intensity ratios of activated U2OS MK2-EGFP cells after incubation with increasing concentrations (0 µM –100 µM) of SB203580 (blue) or Skepinone-L (red) Incubation with Skepinone-L induces an increased nucleocytoplasmic ratio of <1 up to 10 nM of compound concentration indicating a strong inhibitory effect on p38 MAPK. (B) Representative cellular images of the selected data points (scale bar: 20 µm).

Comparing the classical imidazole-based p38 inhibitor SB203580 and the dibenzosuberone based Skepinone-L, we observed a steadily decline of cellular effectiveness of SB203580 in log-dilution experiments. On the contrary, our results show that Skepinone-L inhibits p38 from 10 nM –1 µM in living cells (Figure 5 A and B), probably due to an Gly110 peptide flip binding mode that involves the small gate keeper with a carbonyl oxygen, as found in the X-ray crystal structure [34].

### Selectivity Screening

As JNK3 (c-Jun N-terminal kinase) is a related kinase of p38 MAPK with similar upstream activation mechanisms, we tested the identified top 10 compounds for JNK3 inhibition. As mentioned above, gaining selectivity between p38α and JNK3 is a challenging task. One possible approach to distinguish between those two enzymes is utilizing the different gate keeper residues for p38α and JNK3, which are Thr106 and Met146 respectively. Consequently, as methionine features a bulkier amino acid residue, the overall shape of JNK3’s hydrophobic region I provides less space for residues of inhibitor molecules as p38α does. The binding of inhibitor molecules with bulkier residues that are directed towards entering the hydrophobic region I is therefore unfavorable. For this reason, the hydrophobic region I is also known as “selectivity pocket” in order to distinguish between p38α and JNK3 inhibition.

**Figure 6:**
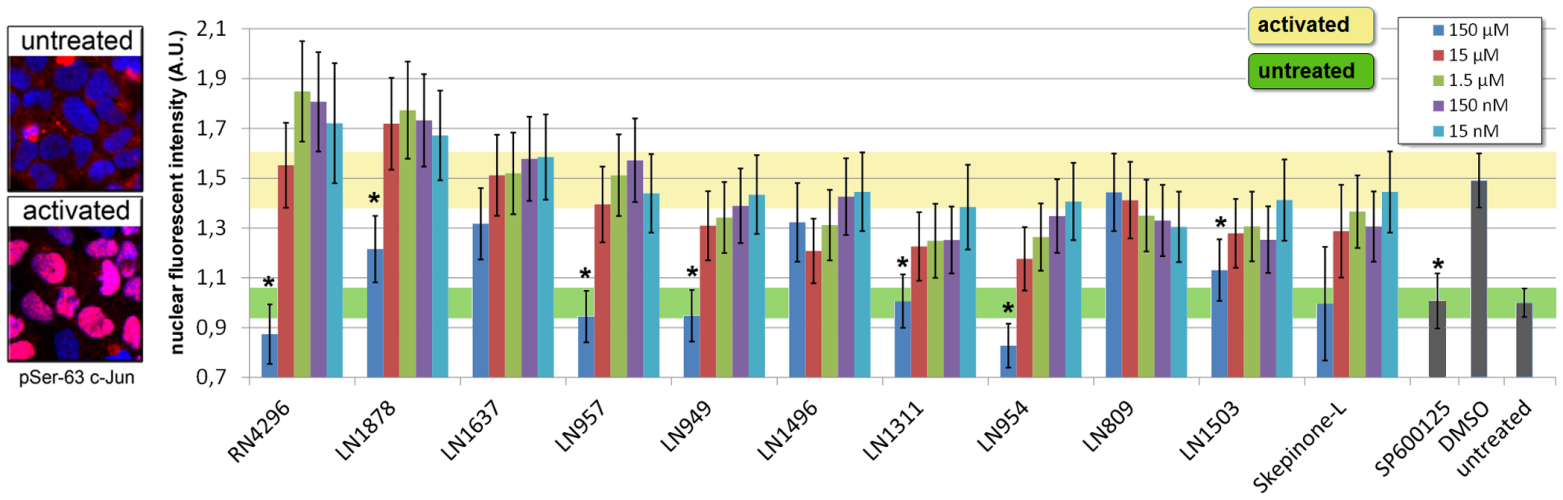
Phospho-Ser63-c-Jun/JNK kinase assay in living cells. (Left) representative pictures of phospho-Ser63-c-Jun antibody signal in the nucleus upon pathway activation. (Right) Top ten lead inhibitors identified from the MK2-EGFP translocation screen have negligible effects on JNK/phosphor-c-jun activity at relevant concentrations (<15 µM). Significant effects (* p<0.05) for several p38 inhibitors are detected at very high concentrations (> = 150 µM). As positive control a specific JNK inhibitor SP600125 is shown. SD derived from 3 independent experiments.

We use an endpoint assay to detect phosphorylation of c-Jun at Ser-63 - a JNK3 specific substrate and component of the AP-1 heterodimer. Selective inhibition of JNK3 activity could be achieved by adding SP600125 - an anthrapyrazolone type JNK3 inhibitor - which served as a positive control. Treatment with SB203580 resulted in dual inhibition of p38 and JNK3 whereas Skepinone-L only inhibited p38 in all relevant dosages proving the excellence performance and zero cross reactivity (data not shown). Screening of the selected top 10 inhibitors in a cellular c-Jun/JNK3-assay show that inhibition of JNK3 activity could only be detected at very high concentrations (150 µM). None of the selected substances showed JNK3 inhibition at physiological relevant concentrations (< = 15 µM) (Figure 6).

### Determination of Cellular Toxicity

**Figure 7:**
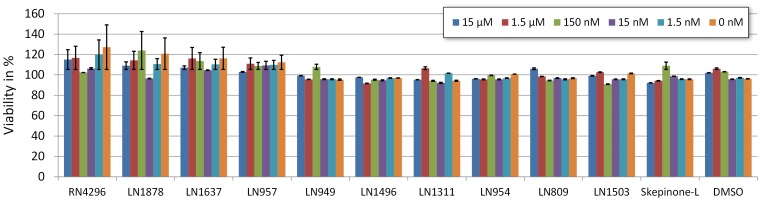
Determination of cellular toxicity of top ten lead inhibitors. Analysis of metabolic viabilities using the AlamarBlue assay after incubation of the cells with the top ten p38 inhibitors for 24 µM. Incubation with DMSO serves as negative control. Shown is standard deviation derived from 3 independent experiments.

Finally we tested whether the compounds have an overall impact on cellular viability upon long term (24 h) incubation. To assess the cellular toxicity of the identified lead compounds we subjected them to resazurin based cell viability and health assay, also known as AlamarBlue. In this assay cells were cells grown in culture media supplemented with different inhibitor concentrations. 24 h after incubation we measured the reduction of the blue colored resazurin to the red fluorescent resorfurin. This is an indicator of the metabolic activity, relevant to predict the toxicological impact of the added compounds. Our results show that treatment of the U2OS MK2-EGFP cells for 24 h with the top 10 lead compounds revealed no cytotoxic effects at any tested concentration levels (15 nM–15 µM) (Figure 7).

## Conclusion

In summary, we present a comprehensive evaluation of a compound library based on cellular screening in combination with biochemical studies and human whole blood assays. The obtained scored table of chemo-structures can now be used to identify the most promising hits for lead optimization. Moreover, integrated data analysis shows the potential of our cell based approach to select hits derived from biochemical assays for further validation in human whole blood assays or animal testings.

In our study we confirmed the outstanding potency of Skepinone-L on cellular accessibility and selectivity [34]. Although Skepinone-L do not affect cellular viability as shown in our assay, we cannot predict the outcome of subsequent animal or human studies. However, based on the results of the cell based MK2-EGFP translocation screen we can provide a set of alternative compounds for future developments. The high selectivity of the identified compounds has many advantages, however, many signaling mechanism related to inflammation in organisms also involve JNK pathways and its downstream effectors. Therefore a therapeutic treatment regimen in many inflammatory diseases might involve a co-inhibition strategy of p38 and JNK based on a combination of optimized dibenzosuberones, or e.g. improved imidazoles subtypes identified here, with specific anthrapyrazolones subtypes or advanced bentamapimods [35], to achieve a sustainable therapeutic-feature and efficacy in preclinical and clinical research.

## Supporting Information

Figure S1 Activation of U2OS MK2-EGFP cell line. Hyperosmotic treatment of the generated U2OS MK2-EGFP RA#2 cell line to establish optimized conditions for the MK2-EGFP translocation assay. Shown are the percentages of activated cells (nucleocytoplasmic ratio <0.7) upon addition of increasing concentration of NaCl either in the presence of DMSO (control) or after addition of two concentrations of the p38 inhibitor SB203580.
(DOCX)

Figure S2 Nucleocytoplasmic ratios after compound treatment. Activated U2OS MK2-EGFP cells were either treated with DMSO or incubated with the p38 inhibitors SB203580 or Skepinone-L (15 µM each) respectively. Shown is the result of four independent experiments, including the standard deviation.
(DOCX)

Table S1 Results of the U2OS MK2-EGFP translocation screen at 15 µM compound concentration. (XLSX)

Movie S1 Visualization of MK2-EGFP translocation after activation of p38 MAPK in real time. U2OS cells stably expressing MK2-EGFP were monitored for 90 min by fluorescent imaging at 3 min time intervals. *Upper section:* Cells were either activated by addition of hyperosmotic media [left] or treated with inhibitor (SB203580 (10 µM) [right]. *Lower section*: Cells were either activated by addition of hyperosmotic media immediately followed by treatment with p38 MAPK inhibitor (SB203580; 10 µM) [left] or incubated with DMSO (0.1 mM) [right].
(MP4)
