# Supplementary material for: A p38 Substrate-Specific MK2-EGFP Translocation Assay for Identification and Validation of New p38 Inhibitors in Living Cells: A Comprising Alternative for Acquisition of Cellular p38 Inhibition Data
Source: PLoS One. 2014 Apr 17;9(4):e95641. doi: 10.1371/journal.pone.0095641 (PMC3990705; doi:10.1371/journal.pone.0095641)
Supplement: Figure S1 — Hyperosmotic treatment of the generated U2OS MK2-EGFP RA#2 cell line to establish optimized conditions for the MK2-EGFP translocation assay. Shown are the percentages of activated cells (nucleocytoplasmic ratio <0.7) upon addition of increasing concentration of NaCl either in the presence of DMSO (control) or after addition of two concentrations of the p38 inhibitor SB203580. (DOCX) [file pone.0095641.s001.docx]

**Supplementary Figure 1**

**Supplementary Figure 1** Hyperosmotic treatment of the generated U2OS MK2-EGFP RA#2 cell line to establish optimized conditions for the MK2-EGFP translocation assay (z’ values >0.5 at 300-400 mOsm; NaCl supplementation). Shown are the percentages of activated cells (nucleocytoplasmic ratio < 0.7) upon addition of increasing concentration of NaCl either in the prescence of DMSO (control) or after addition of two concentrations of the SB203580 (p38 inhibitor)
