# Supplementary material for: A p38 Substrate-Specific MK2-EGFP Translocation Assay for Identification and Validation of New p38 Inhibitors in Living Cells: A Comprising Alternative for Acquisition of Cellular p38 Inhibition Data
Source: PLoS One. 2014 Apr 17;9(4):e95641. doi: 10.1371/journal.pone.0095641 (PMC3990705; doi:10.1371/journal.pone.0095641)
Supplement: Figure S2 — Activated U2OS MK2-EGFP cells were either treated with DMSO or incubated with the p38 inhibitors SB203580 or Skepinone-L (15 µM each) respectively. Shown is the result of four independent experiments, including the standard deviation. (DOCX) [file pone.0095641.s002.docx]

**Supplementary figure 2:**

**supplementary figure 2:** Determination of nucleocytoplasmic ratios of activated U2OS MK2-EGFP RA#2 cells after incubation with DMSO or with SB203580 or Skepinone-L (15 µM each). Shown is standard deviation of 4 independent experiments
